# Population-specific, recent positive directional selection suggests adaptation of human male reproductive genes to different environmental conditions

**DOI:** 10.1186/s12862-019-1575-0

**Published:** 2020-02-13

**Authors:** Helmut Schaschl, Bernard Wallner

**Affiliations:** 1 Department of Evolutionary Anthropology, University of Vienna, Althanstrasse 14, 1090 Vienna, Austria; 2 Department of Behavioural Biology, University of Vienna, Althanstrasse 14, 1090 Vienna, Austria

**Keywords:** Human testis-enriched genes, Male reproductive genes, Genetic adaption, Positive selection

## Abstract

**Background:**

Recent human transcriptomic analyses revealed a very large number of testis-enriched genes, many of which are involved in spermatogenesis. This comprehensive transcriptomic data lead us to the question whether positive selection was a decisive force influencing the evolution and variability of testis-enriched genes in humans. We used two methodological approaches to detect different levels of positive selection, namely episodic positive diversifying selection (i.e., past selection) in the human lineage within primate phylogeny, potentially driven by sperm competition, and recent positive directional selection in contemporary human populations, which would indicate adaptation to different environments.

**Results:**

In the human lineage (after correction for multiple testing) we found that only the gene *TULP2*, for which no functional data are yet available, is subject to episodic positive diversifying selection. Using less stringent statistical criteria (uncorrected *p*-values), also the gene *SPATA16*, which has a pivotal role in male fertility and for which episodes of adaptive evolution have been suggested, also displays a putative signal of diversifying selection in the human branch. At the same time, we found evidence for recent positive directional selection acting on several human testis-enriched genes (*MORC1*, *SLC9B1*, *ROPN1L*, *DMRT1*, *PLCZ1*, *RNF17*, *FAM71D* and *WBP2NL*) that play important roles in human spermatogenesis and fertilization. Most of these genes are population-specifically under positive selection.

**Conclusion:**

Episodic diversifying selection, possibly driven by sperm competition, was not an important force driving the evolution of testis-enriched genes in the human lineage. Population-specific, recent positive directional selection suggests an adaptation of male reproductive genes to different environmental conditions. Positive selection acts on eQTLS and sQTLs, indicating selective effects on important gene regulatory functions. In particular, the transcriptional diversity regulated by sQTLs in testis-enriched genes may be important for spermatocytes to respond to environmental and physiological stress.

## Background

The remarkable diversity of life histories is inevitably linked to the optimisation of the reproductive system in species. In evolutionary biology, the important question is therefore what role natural selection has played in the evolution of the reproductive systems in different species. The key male reproductive organ in humans is the testes. They have two main functions: the efficient production of sperm (spermatogenesis) over a male’s reproductive life span and the synthesis of hormones necessary to develop male sex characteristics. Spermatogenesis takes place in the testis within the seminiferous tubules, supported by Sertoli cells. This process comprises highly complex cellular events in which the proliferation and maturation of germ cells, derived from self-renewing stem cells, produces about 200 million sperm daily from puberty through the entire male adulthood [1]. Human spermatogenesis requires about 70 days for a complete cycle. Due to the very high number of mitotic replications of spermatogonia and the subsequent critical reduction of chromosome number in spermatocytes to the haploid state, the male reproductive system needs to maintain and protect the genomic integrity in the spermatocytes against the accumulation of DNA replication errors and exposure to environmental mutagens. The second important function of the testes is steroidogenesis within the Leydig cells, where cholesterol is converted to testosterone. Testosterone, together with the two gonadotropic hormones follicle stimulating-hormone (FSH) and luteinizing hormone (LH) form the testicular endocrinal system that controls spermatogenesis and the development of sexual characteristics. The mature and ejaculated spermatozoa are carried to the female tract in seminal plasma, which supports key sperm functions such as interactions with the various environments of the tubular genital tract, with the oocyte and with the female immune system and potentially helps modulate sperm rejection or tolerance [2].

Recent tissue-specific transcriptomic analyses of humans revealed a very large number of expressed genes in the testis [3–5]. The Human Protein Atlas database (www.proteinatlas.org) reports that about 84% (*n* = 16,598) of all human proteins are expressed in this tissue, and about 950 of these genes show testis-enriched expression when compared with all other analysed human tissues. Testes therefore belong to the tissues (like the brain) with the largest number of tissue-enriched genes. Many of the testis-enriched genes are related to testis-specific functions and spermatogenesis [3, 4].

These comprehensive transcriptomic data raise the question whether episodic positive diversifying selection was a decisive force influencing the evolution and variability of the testis-enriched genes in the human lineage. The selective pressures on the amino-acid level can be quantified by models of molecular evolution that incorporate the ratio (*ω*) of nonsynonymous (*d*_N_) to synonymous (*d*_S_) substitutions within and among species [6]. The ratio (*ω*) can vary over sites (site-to-site) and time (branch-site). Branch-site models enable studying the history of natural selection under particular phylogenetic hypotheses by measuring *ω* in different lineages along the phylogeny. If changes in amino acids offer selective advantages, leading to accelerated fixation of the nonsynonymous mutations, then the nonsynonymous substitution rate will be higher than the synonymous rate (*ω* = *d*_N_/*d*_S_ > 1). This would indicate positive diversifying selection. If *ω* < 1, then negative selection can be inferred, while *ω* = 1 suggests that the protein is evolving neutrally [7–9].

We should expect, however, that the coding sequences of important reproductive genes are mostly under purifying selection. This is because nonsynonymous substitutions may alter the structure of a protein and therefore harm its function and consequently fitness. Accordingly, phenotypic differences between closely related species or populations should be driven rather by gene regulatory changes, such as cis-regulatory elements (e.g., promoters, enhancers etc.), than by changes in the coding sequences. Nonetheless, a significant number of male fertilization genes show accelerated evolution in the coding sequences in different species (reviewed by [10]). This has led to the question why the rapid evolution of reproductive proteins is a widespread phenomenon. Several mechanisms such as sperm competition, pathogen resistance, cryptic female choice, sexual conflict, reinforcement, and avoidance of heterospecific fertilization have been forwarded [10, 11]. In particular, sperm competition, in which ejaculates from more than one male compete for the fertilization of a female’s eggs, is thought to be a powerful mechanism of (post-copulatory) sexual selection. This is because it can potentially generate selective pressure to increase testis size and sperm numbers, to change sperm phenotype to increase swimming speed, and to alter male physiology [12–18]. In primates, the expressed proteins of *protamine P1* (*PRM1*) and *protamine P2* (*PRM2*) are the most abundant sperm nuclear proteins and play a crucial role in correctly packaging the paternal DNA. *PRM1* and *PRM2* are two of the most rapidly diverging proteins in some primate species [19]. Subsequent studies found that the rapid evolution of protamine genes in humans and chimpanzees is due the action of positive selection, which is possibly linked to sperm competition [18, 20]. Furthermore, several studies report accelerated evolution of different male reproductive genes in human and non-human primates, including the genes *spermatogenesis associated 16* (*SPATA16*) [21], *ESX homeobox 1* (*ESX1*) [22], *zonadhesin* (*ZAN*) [23], *polycystin family receptor for egg jelly* (*PKDREJ*) [24], and *semenogelin 2* (*SEMG2*) [25, 26]. These genes are functionally involved in spermatogenesis, and positive selection at these genes is thought to be driven mainly by sperm competition.

In contrast to the codon-substitution model, which detects past selection, population genetics models of natural selection detect ongoing selection in populations. Modern humans spread from Africa within about the last 80,000 years to different parts of the world and populated a remarkably broad range of environments. Moreover, during the Neolithic demographic transition about 9000 to 13,000 years ago most humans switched from being hunter-gatherers to agriculturists, which included substantial changes in lifestyles associated with plant and animal domestication. Contemporary humans not only inhabit diverse environments but also display a wide phenotypic diversity across geographically distributed populations; much of this diversity undoubtedly reflects genetic adaptation to the different environmental conditions [27]. Whether any of the human testis-enriched expressed genes show a signature of recent positive directional selection, which would indicate adaptation to different environments, has not yet been studied comprehensively incorporating the recently available extensive transcriptomic data.

In the present study, we used two methodological approaches to detect different levels of positive selection, namely episodic positive diversifying selection (i.e., past selection) in the human lineage within the primate phylogeny and recent positive directional selection in contemporary human populations. Specifically, we used the recently published method by Smith and co-workers [28], the adaptive branch-site random effects likelihood method (aBSREL), to test the hypothesis that episodic positive diversifying selection in the human lineage acted on testis-enriched genes, in particular on genes involved in spermatogenesis, possibly driven by sperm competition. Furthermore, we applied the integrated haplotype score method (iHS) [29] to identify human testis-enriched genes that are under recent positive directional selection in diverse human populations, which would indicate local genetic adaptation to different environments.

## Methods

### Human testis-specific transcriptome data

We obtained the testis-specific transcriptome/proteome data from the Human Protein Atlas database (https://www.proteinatlas.org/humanproteome/tissue/testis) [5]. In total, 950 genes are testis-enriched expressed, showing an at least four-fold higher mRNA level in the testes compared to any other tissues. The data were accessed and downloaded between April and September 2019.

### Human 1000 genomes project phase 3 SNP data

We used the phased genetic data of the 1000 Genomes project phase 3 data (FTP server: http://ftp.1000genomes.ebi.ac.uk/vol1/ftp/release/20130502/). We included from this database single nucleotide polymorphism (SNP) data from 12 human populations with the following genetic ancestries (as defined by the 1000 Genome Project) and number of subjects (n): East Asian ancestry: Han Chinese in Bejing, China (CHB, *n* = 103)), Japanese in Tokyo, Japan (JPT, *n* = 104), and Kinh in Ho Chi Minh City, Vietnam (KHV, *n* = 99); South Asian ancestry: Bengali in Bangladesh (BEB, *n* = 86), Indian Telugu in the United Kingdom (ITU, *n* = 102) and Punjabi in Lahore, Pakistan (PJL, *n* = 96)); African ancestry: Gambians in Western Division, The Gambia (GWD, *n* = 113), Luhya in Webuye, Kenya (LWK, *n* = 99), and Esan in Nigeria (ESN, *n* = 99)); European ancestry: British in England and Scotland, United Kingdom (GBR, *n* = 91), Finnish in Finland (FIN, *n* = 99), and Toscani in Italy (TSI, *n* = 99). Because of the underlying population genetics models of natural selection, we excluded recently admixed populations and populations that are in close geographic proximity. We used the software programmes PLINK 1.9 [30] (https://www.cog-genomics.org/plink/1.9/ and VCFtool v0.1.14 [31] (https://vcftools.github.io/index.html) to process variant call format (VCF) files from the 1000 Genomes database for all chromosomes. We also excluded all structural variants and restricted our analysis to bi-allelic SNPs with minor allele frequency (MAF) > 0.05. The UCSC Genome Browser (http://genome.ucsc.edu/) was used to retrieve the genomic position of the testis-specific genes (including 5kbp up- and downstream of the gene) in accordance to the reference genome GRCh37/hg19.

### Phylogeny selection for lineage-specific analysis

We used the software BioMart [32], which is integrated in the Ensembl database [33] (http://www.ensembl.org), to obtain the human DNA gene sequences of the human testis-enriched genes as well as the corresponding orthologous genes of chimpanzee (*Pan troglodytes*), gorilla (*Gorilla gorilla*), orang-utan (*Pongo abelii*), macaque (*Macaca mulatta*), olive baboon (*Papio anubis*), and common marmoset (*Callithrix jacchus*). The primate species studied also present different mating systems and testis sizes [34]. We used the Basic Local Alignment Search Tool (BLAST) (https://blast.ncbi.nlm.nih.gov/Blast.cgi), biomaRt version 2.40.0 within the R version 3.5/Bioconductor programme [35], as well as a python script to obtain the DNA sequences from orthologous genes from GenBank (https://www.ncbi.nlm.nih.gov/genbank/) [36]. We included in the evolutionary analysis only testis-enriched genes that showed *d*_N_/*d*_S_ ≥ 2.0 on the Ensembl database, i.e., human sequences vs. the other orthologous primate genes, and genes known to be under positive selection in primate branches. In total, we analysed 87 human testis-specific genes for episodic positive diversifying selection in the subsequent evolutionary analysis. The software programme AliView version 1.26 [37] with the integrated alignment programme MUSCLE version 3.8.425 [38] was used to generate codon-based alignments of the gene sequences. The few cases where no homologous gene sequences were available or could not be properly aligned were excluded from the analysis.

### Evolutionary analysis: detection of episodic positive diversifying selection in the human lineage

We used the adaptive branch-site random effects likelihood (aBSREL) method to identify human testis-enriched genes that show signs of episodic positive diversifying selection [28]. The method models both the site-level and branch-level *ω* distribution over sites, and tests for each branch in the phylogeny whether a proportion of sites have evolved under positive selection. The method acknowledges that different branches may feature more or less complex evolutionary patterns and hence may be better modelled by more or fewer *ω* classes. Significance was assessed by the likelihood ratio test (LRT) at a threshold of *p* ≤ 0.05. The aBSREL method uses the implemented Holm–Bonferroni sequential rejection procedure to control the family-wise error rate [28]. In this study, however, we report both the corrected test *p*-values and the uncorrected *p*-values. The aBSREL is implemented and available from the Datamonkey.org webserver (http://www.datamonkey.org/absrel) [39].

### Population genetic analysis: detection of positive selection and F_ST_ analysis

We used the integrated haplotype score test (iHS) to detect genome-wide positive selection [29]. The iHS approach compares integrated EHH (Extended Haplotype Homozygosity) values between alleles at a given SNP; the method is based on the decay of haplotype homozygosity as a function of recombination distance. The underlying rationale is that selected alleles will have an unusually long-range linkage disequilibrium (LD) given their frequency in the population. Significant negative iHS values (absolute iHS score < − 2.0) indicate unusually long haplotypes carrying the derived allele, and significant positive values (absolute iHS score > 2.0) are associated with long haplotypes carrying the ancestral allele [29]. We used the software programme selscan version 1.2.0a (https://github.com/szpiech/selscan), which has implemented the iHS/EHH approaches [40], to analyze the genomic data for sites under positive selection. All scans were run on phased whole chromosome data with the default model parameters of the selscan programme. The unstandardized iHS scores were normalized in frequency bins across the entire genome using the script *norm*, provided with the selscan programme. We considered a SNP to have a candidate selection signal if it was within a ‘cluster’ of ≥20 SNPs that also had elevated iHS scores. We used a bash script to identify, among the 950 testis-enriched genes, those that showed evidence for positive directional selection in at least three populations per genetic ancestry, i.e., in Africans (AFR), Europeans (EUR), South Asians (SAS), and East Asians (EAS). In addition, we used the R package REHH to analyse the data and to generate outputs of the EHH decay plots [41]. Pairwise *F*_*ST*_ were calculated for each SNP under positive selection using the Weir & Cockerham *F*_*ST*_ calculation [42], which is implemented in VCFtool v0.1.14 programme [31].

### Gene ontology (GO) analysis and genotype-tissue expression (GTEx) data

The GO molecular function and biological process of the studied genes were obtained from neXtProt release 2019-01-11 [43, 44]. Furthermore, we used the open-source GOnet web-application (available at http://tools.dice-database.org/GOnet/) to perform GO term annotation analysis and graphical presentation of the human genes found to be under positive selection [45]. The GTEx Portal V8 Release (https://www.gtexportal.org/home/) was used to obtain data (dbGaP Accession phs000424.v8.p2) on expression quantitative trait loci (eQTLs) and splicing quantitative trait loci (sQTLs) [46].

## Results

### Positive diversifying selection of testis-enriched genes in the human lineage

Previous studies found that the genes *PRM1*, *PRM2*, *ESX1*, *SPATA16*, *CATSPER1*, *ZAN*, and *PKDREJ* evolve rapidly in the human lineage [18, 20–26]. We first used the branch-site aBSREL method to reanalyse these genes to find evidence of positive diversifying selection in the human branch. The original hypothesis that these genes in the human lineage are under positive selection was not supported by the aBSREL analysis because the human branches had, after correction for multiple testing, test *p*-values > 0.05. Accordingly, the null hypothesis of neutral or negative selection is not rejected for these genes (Additional file 1). Among the other analysed testis-enriched genes, after correction for multiple testing, only the gene *tubby like protein 2* (*TULP2*) remains significantly (test *p*-value = 0.027) associated with positive diversifying selection in the human branch (Table 1). However, if we consider the uncorrected *p*-values (at the threshold ≤0.05), then aBSREL also identifies the genes *C9orf43*, *C9orf131*, *C12orf40*, *FAM209A*, *MAGEB16*, *NACA2*, *POTED*, *SPATA16*, *TMCO5A*, and *ZFAND4* as potential candidates for such selection (Table 1). Few biological data are available for most of these genes. The GO analysis and the literature suggest that the proteins of the *SPATA16* and possibly of *TMCO5A* and *MAGEB16* are involved in spermatogenesis [47–51]. Furthermore, the *POTED* gene belongs to the primate-specific *POTE* gene family. The genes of this family are expressed in spermatids and the expressed proteins potentially play a role in cell apoptosis [52].

**Table 1:** Results of the aBSREL analysis with the *ω* distribution over the sites of the human testis-enriched genes with corrected and uncorrected *p*-values (in bold, the significant test *p*-value). The gene ontology terms (GO) are also given

Gene	Chr	Gene description	GO molecular/GO biological	Test *p*-value	Uncorrected *p*-value	*ω* distribution over sites
*SPATA16*	3	Spermatogenesis associated 16	Spermatogenesis GO:0007283 Cell differentiation GO:0030154	0.21	0.019	*ω*1 > 1000 (100%)
*C9orf43*	9	Chromosome 9 open reading frame 43	Protein binding GO:0005515	0.21	0.026	*ω*1 > 1000 (100%)
*C9orf131*	9	Chromosome 9 open reading frame 131	No data available	0.11	0.012	*ω*1 = 7.19 (100%)
*ZFAND4*	10	Zinc finger AN1-type containing 4	Zinc ion binding GO:0008270	0.55	0.05	*ω*1 = 1000 (100%)
*C12orf40*	12	Chromosome 12 open reading frame 40	Protein binding GO:0005515	0.35	0.032	*ω*1 > 1000 (100%)
*TMCO5A*	15	Transmembrane and coiled-coil domains 5A	No data available	0.4	0.036	ω1 > 1000 (100%)
*NACA2*	17	Nascent polypeptide associated complex alpha subunit 2	Protein transport GO:0015031	0.4	0.044	*ω*1 > 1000 (100%)
***TULP2***	19	**Tubby like protein 2**	Phosphatidylinositol binding GO:0035091 Protein-containing complex binding GO:0044877 Protein localization to cilium GO:0061512 Protein localization to photoreceptor outer segment GO:1903546 Visual perception GO:0007601	**0.027**	**0.002**	***ω*1 = 0.00 (98%)** ***ω*2 = 1290 (1.5%)**
*FAM209A*	20	Family with sequence similarity 209 member A	No data available	0.07	0.008	*ω*1 = 0.00 (96%) ω2 = 1350 (4.0%)
*POTED*	21	POTE ankyrin domain family member D	Actin binding GO:0003779 Actin cytoskeleton organization GO:0030036	0.098	0.011	*ω*_1_ > 1000 (100%)
*MAGEB16*	X	MAGE family member B16	No data available	0.4	0.044	*ω*1 = 0.00 (97%) *ω*2 = 42.3 (3.3%)

### Positive diversifying selection of testis-specific genes in non-human primate lineages

The branch-site method (aBSREL) found evidence (test *p*-value ≤0.05) of positive diversifying selection in 12 out of 87 analysed orthologous testis-specific genes in the non-human primate lineages (Additional file 1). Most genes show a species-specific signature of diversifying selection (Additional file 2). The GO analysis did not yield any significantly enriched pathways. Other, functional studies, however, suggest that some of these genes are involved in spermatogenesis and fertilization. The expressed proteins of *SEMG2* are involved in the formation of the semen coagulum [25, 53]. This gene has already been found to be subjected to positive diversifying selection in the chimpanzee lineage and in the white-cheeked gibbon lineage [25, 26]. We determined here that this gene in the marmoset lineage is subjected to positive diversifying selection. In this species, the gene *AKAP4* also shows a signature of such selection. For this gene, a recent functional genetic study on mice showed its indispensable role in the integrity of the sperm flagellum and in spermatozoa maturation [54]. Furthermore, we identified the gene *INHA*, which is functionally involved in regulating follicle-stimulating hormone secretion [55], to be subjected to diversifying selection in the Rhesus macaque and olive baboon.

### Positive selection of testis-enriched genes in different human populations

**Fig. 1:**
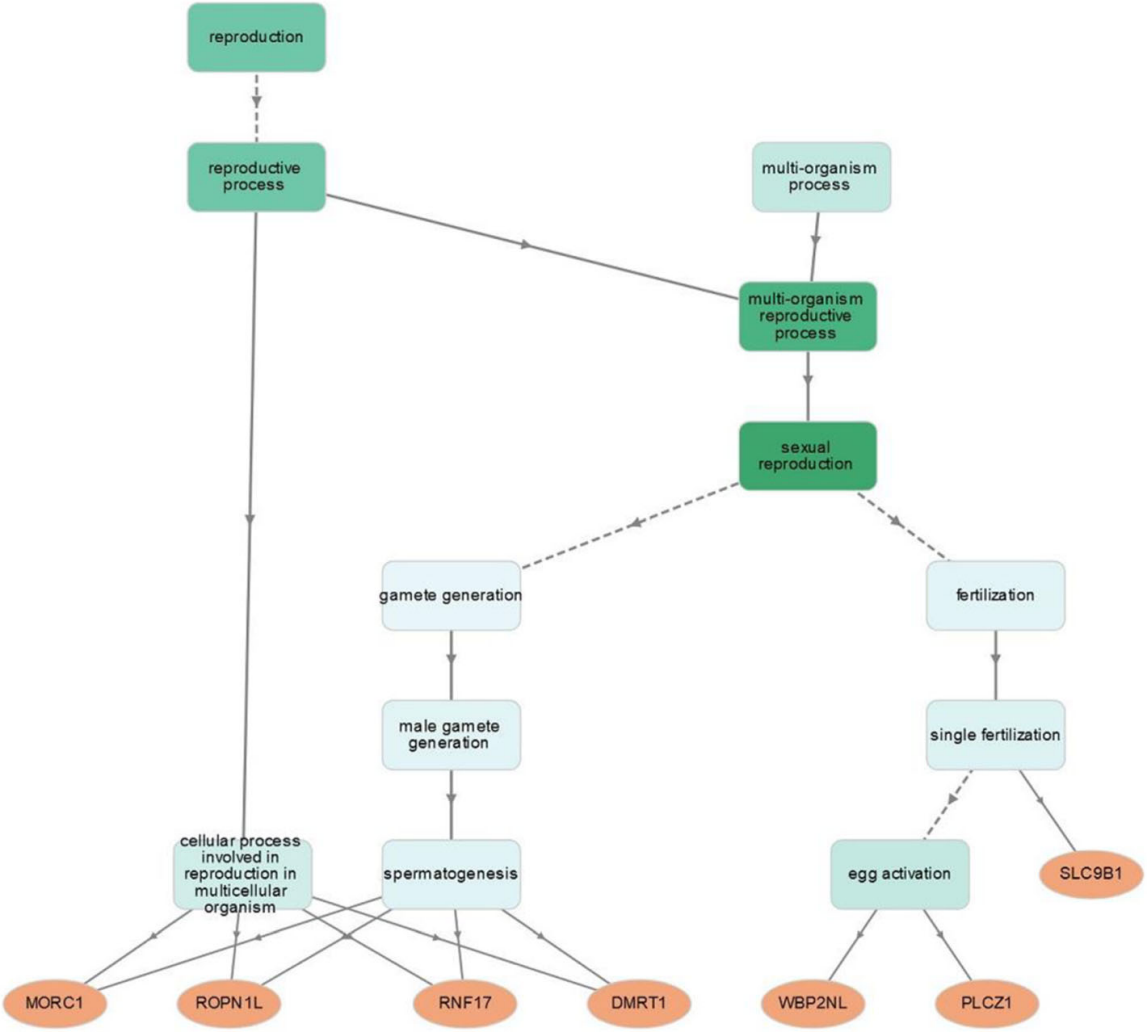
Graphical presentation of the significant (*p* < 4.12e-5) GO terms for testis-enriched genes under positive selection in hierarchical layout (less specific GO terms are placed at the top of the network, more specific GO terms at the bottom)

The LD-based test statistics iHS detected several testis-enriched genes under recent positive directional selection (Table 2). In the populations with African genetic ancestry, the genes *MORC1*, *RNF17,* and *WBP2NL* are under positive selection. In Europeans, this also appears to be the case for *FAM71D* as well as *DMRT1* and *PLCZ1*; the latter two are also positively selected in South Asians. In East Asians, only the gene *ROPN1L* is under positive selection. The solute carrier *SLC9B1* is positively selected in all studied human populations. However, this selection acts on this gene in Africans on ancestral alleles, whereas in the non-African populations the derived alleles show a signature of positive selection (Additional file 3). The gene enrichment analysis shows that the genes under selection are involved in spermatogenesis (*DMRT1*, *MORC1*, *RNF17*, *ROPN1L*), in egg activation (*PLCZ1* and *WBP2NL*) and single fertilization (zygote formation) (*SLC9B1*) (Fig. 1). We obtained no GO terms for *FAM71D*, but a recent functional genetic study revealed that *FAM71D* is expressed in the flagellum of mature sperm in both mice and humans [56]. The two SNPs rs3974604 and rs11722779 of the gene *SLC9B1* that are under positive selection are associated with variation in isoform usage (splicing quantitative trait loci – sQTL) (Additional file 4). These SNPs also show relative high pairwise *F*_*ST*_ (> 0.28) between the African populations and the other continental groups (Additional file 5). Finally, the SNP rs71431709 of *RNF17*, which is under positive selection only in Africans, also presents a sQTL (Additional file 4). The SNP rs10459068 of the *PLCZ1* gene, which is under positive selection in Europeans and South Asians, functions as an expression quantitative trait locus (eQTL), and the derived-T allele of this SNP is associated with increased gene expression (Additional file 6).

**Table 2:** Human testis-enriched genes under positive selection detected in different human populations and genetic ancestries. Given are the SNPs with the highest iHS values, gene ontology (GO) terms and available QTL information (from the Genotype-Tissue Expression (GTX) database)

Gene	Chr	Gene description	GO molecular function/GO biological process	Genetic ancestry	iHS	GTEx testis tissue
*MORC1*	3	MORC family CW-type zinc finger 1	DNA methylation involved in gamete generation GO:0043046 Zinc ion binding GO:0008270 Spermatogenesis GO:0007283 DNA hypermethylation GO:0044026	AFR	rs12695191: 3.7	–
*SLC9B1*	4	Solute carrier family 9 member B1	Flagellated sperm motility GO:0030317	AFR	rs3974604: 4.1	sQTL
			Proton transmembrane transport GO:1902600	EUR	rs11722779: −4.0	
			Regulation of intracellular pH GO:0051453	SAS	rs11722779: −3.9	sQTL
			Single fertilization GO:0007338	EAS	rs11722779: −3.5	
			Sodium ion transmembrane transport GO:0035725			
*ROPN1L*	5	Rhophilin associated tail protein 1 like	cilium movement GO:0003341 flagellated sperm motility GO:0030317 sperm capacitation GO:0048240	EAS	rs2673855: −2.8	–
*DMRT1*	9	Doublesex and mab-3 related transcription factor 1	Transcription regulator activity GO:0140110 Sex determination GO:0007530 Developmental process involved in reproduction GO:0003006 Gamete generation GO:0007276	EUR SAS	rs166790: −3.4 rs166790: −3.1	–
*PLCZ1*	12	Phospholipase C zeta 1	Phosphatidylinositol phospholipase C activity GO:0004435 Phosphatidylinositol-3-phosphate binding GO:0032266 Egg activation GO:0007343 Positive regulation of cytosolic calcium ion concentration involved in egg activation GO:0060470	EUR SAS	rs10459068: −2.7 rs10459068: −2.7	eQTL
*RNF17*	13	Ring finger protein 17	Metal ion binding GO:0046872 Spermatid development GO:0007286	AFR	rs71431709: 2.7	sQTL
*FAM71D*	14	Family with sequence similarity 71 member D	No data available	EUR	rs10431714: −3.4	–
*WBP2NL*	22	WBP2 N-terminal like	Chromatin DNA binding GO:0031490 WW domain binding GO:0050699 Transcription coactivator activity GO:0003713 Meiotic cell cycle GO:0051321 female pronucleus assembly GO:0035038 male pronucleus assembly GO:0035039	AFR	rs57796605: 3.0	–

## Discussion

### Episodic positive diversifying selection in the human lineage

Our study found little evidence for widespread episodic positive diversifying selection in the human lineage. After correction for false discovery rates, only the gene *TULP2* remained statistically significantly (test *p*-value = 0.027) associated with diversifying selection. The exact function of this gene is not known yet. It does, however, appear to also be expressed in the human retina [57]. It is therefore unclear whether this form of selection acting on *TULP2* is linked to its function in the retina or in the testis.

Furthermore, aBSREL found evidence of positive diversifying selection for 12 testis-enriched, orthologous genes in non-human primates. The GO analysis revealed an association with reproduction only for *SEMG2* (flagellated sperm motility and sperm capacitation), *AKAP4* (spermatogenesis) and *INHA* (positive regulation of follicle-stimulating hormone secretion). In addition, our study provides evidence that *RHOXF2*, an X-linked homeobox gene, exhibits diversifying selection in the chimpanzee lineage, confirming a previous study that showed strong positive selection for the lineages leading to humans and chimpanzees [58]. We found *SEMG2* to be subjected to positive diversifying selection in the common marmoset lineage, as previously reported for the chimpanzee and white-cheeked gibbon lineages [25, 26]. The chimpanzee has a multi-male mating system, and the common marmoset breeding system is flexible, ranging from monogamous and polygynous to polyandrous [59]. It is therefore currently not possible to draw conclusions about the impact of different mating systems and thus potential sperm competition on this gene in these species.

If we accept less stringent statistical criteria, i.e., using the uncorrected *p*-values at the threshold ≤0.05, then for the human lineage several other human testis-enriched genes show a potential signature of diversifying selection (see Table 1). For most of these genes, however, no comprehensive biological data are available. For example, the gene *SPATA16* – for which episodes of adaptive evolution in both the human and the chimpanzee lineage have been suggested [21] – displays a putative signal of diversifying selection (albeit only in the human branch in our study). Functional genetic studies suggest that the *SPATA16* molecules play important roles in human sperm formation and male fertility [51, 60]. Recent studies suggest that at least *MAGEB16* is potentially involved in spermatogenesis [48, 50], and possibly *TMCO5A*, as shown in the rat model [49]. Furthermore, *POTED* belongs to the primate-specific *POTE* gene family. The POTE proteins have a pro-apoptotic function, and these proteins are highly expressed in human round spermatids that are undergoing apoptosis [52]. Nonetheless, these genes are not statistically substantiated (after correction for multiple testing), so that it remains speculative whether they have actually evolved under diversifying selection in the human lineage.

Why have not we found the same human testis-specific genes to be under positive diversifying selection as previous studies? Most of those earlier studies used the branch-site models implemented in the PAML method (Phylogenetic Analysis by Maximum Likelihood) [61], which differs from the method used here. The adaptive branch-site method aBSREL analyses the data under a model whose complexity is inferred from the data together with continuous model parameters [28]. Smith et al. [28] showed that most branches in gene phylogenies can be adequately modelled with a single *ω* ratio model. This greatly reduces model complexity, thereby increasing the sensitivity to detect episodic positive diversifying selection in the phylogenies. Furthermore, most studies that tested more than one branch did not control for the family-wise error rate. In the present study, we therefore applied the implemented Holm–Bonferroni sequential rejection procedure to correct for multiple testing. Apart from the methodological differences, there is also the possibility that the role of diversifying selection in driving male reproductive genes is overestimated. In fact, several studies discussed and suggested that relaxation of purifying selection rather than positive selection is responsible for the fast evolutionary rates found in certain reproductive genes [62–65]. Moreover, because of the stochastic nature of mutation, it is expected that *d*_N_ > *d*_S_ will frequently occur at certain codons merely by chance [62]. Note also that sperm competition has been invoked as an important selective force driving the evolution of some male reproductive genes. Among primates, testis size varies, and several studies suggest an association between relative testis size and mating system in primates and the level of sperm competition. Monogamous or polygynous primates typically have relatively small testes, whereas testis size is relatively large in species with a multi-male system that potentially involves sperm competition (reviewed by [66]). The size of the human testis is intermediate relative to body size, somewhat closer to the monogamous gorilla than the polygamous chimpanzee [13]. This suggests that, in contrast to chimpanzees, humans (like gorillas) may not have been subject to strong positive diversifying selection driven by sperm competition for high levels of ejaculate production [67]. Combining all these results leads us to conclude that this form of selection probably did not play its purportedly important role in the evolution of human male reproductive genes.

### Evidence for positive directional selection in human populations

We found several testis-enriched genes to be under recent positive directional selection in different human populations. In Africans, the genes *MORC1*, *RNF17* and *WBP2NL* are under positive selection. *MORC1* and *RNF17* are involved in spermatogenesis and *WBP2NL* in egg activation. In Europeans, the genes *DMRT1*, *PLCZ1* and *FAM71D* show signatures of positive selection. The expressed protein of the *PLCZ1* gene (PLCζ) plays an important role at oocyte activation. PLCζ localizes in the acrosome in spermatozoa and elicits Ca (2+) oscillations for oocyte activation during fertilization [68]. Moreover, in this gene the derived-T allele of the SNP rs10459068 functions as an eQTL and is associated with increased expression, suggesting that positive selection drives higher expression of this gene in Europeans and South Asians (Additional file 6). The frequency of the derived-T allele also differs substantially between Europeans/South Asians and Africans because the derived allele occurs in Africans at less than 9%, whereas in Europeans and South Asians the frequencies are 56 and 63%, respectively. The gene *FAM71D*, which is under positive selection only in Europeans, is expressed in the flagellum of mature sperm in both mice and humans, suggesting functional involvement in sperm motility [56]. The SNP rs10431714 of this gene shows relative high *F*_*ST*_ values between different continental groups (Additional file 5). For example, Europeans are highly diverged from Africans at this locus, with *F*_*ST*_ = 0.69. In East Asians, *ROPN1L* is under positive selection in a population-specific manner. This gene plays an important role in spermatozoa capacitation and sperm motility [69]. This gene is, however, embedded in a larger genome region that is under positive selection, which also includes the gene membrane-associated ring finger (C3HC4) 6, E3 ubiquitin protein ligase (*MARCH6*). It is therefore unclear whether positive selection is acting mainly on *ROPN1L* or on *MARCH6* in East Asians.

The solute carrier gene *SLC9B1* is under positive selection in all studied populations. This gene belongs to the *SLC9* family of genes that encode Na+/H+ exchangers that play a role in regulating pH, cell volume and ion homeostasis [70–72]. Spermatozoa are exposed in different tissues to different pH levels that increase from a relatively low pH < 7 in the cauda epididymis to pH ~ 7.4 in the female oviduct. Accordingly, intracellular pH regulation is very important for sperm physiology, including motility, maturation and the acrosome reaction [70, 73]. Indeed, experimental studies in animals showed that *SLC9B1* is essential not only for male fertility, but also for survival [70, 71]. This male reproductive gene is probably vital for reproduction in many species. In humans, specific methylated sites within this gene are associated with foetal distress [74]. Finally, this gene and for *RNF17* the positively selected SNPs present splicing QTLs (sQTLs), which are associated with changes in the splicing ratios of the transcripts (Additional file 4). Alternative splicing contributes to transcript diversity, enabling a gene to express different mRNAs and thus encode xdifferent proteins. Positive selection acting on the SNP sQTLs of these two genes may be an important molecular mechanism to generate a broader repertoire of functional isoforms of testis-enriched genes. The functional diversity of testis-enriched transcripts may be particularly important in enabling spermatocytes to respond to environmental and perhaps also to physiological stress such as the above-mentioned exposure to different pH levels.

## Conclusion

We conclude that episodic diversifying selection, possibly driven by sperm competition, was not an important force driving the evolution of testis-enriched genes in the human lineage. However, recent positive directional selection plays an important role for various testis-enriched genes that have vital functions in human reproduction. Almost all genes are population-specifically under positive selection, suggesting genetic adaptation to different environmental conditions. The gene *SLC9B1* is under positive selection in all studied populations, possibly linked to its important function in male fertility. Moreover, positive selection acts on eQTLs and sQTLs, suggesting selective effects on important gene regulatory functions. Functional transcript diversity regulated by sQTLs may be important for spermatocytes to respond to environmental and physiological stress.

## Supplementary information

**Additional file 1: Table S1. ** Results of the adaptive branch-site random effects likelihood (aBSREL) analysis.

**Additional file 2: Table S2.** Results of the aBSREL analysis with the ω distribution over sites of testis-specific genes in non-human primate branches with test *p*-value ≤0.05. Given are gene known ontology (GO) terms for the genes.

**Additional file 3: Figure S1.** EHH plot of the SNP rs11722779 of gene *SLC9B1* in the European (TSI) vs. African (LWK) vs population.

**Additional file 4: Figure S2.** Violin plots showing the SNP splicing QTLs (sQTLs) of the testis-enriched genes *SLC9B1* and *RNF17* that are under positive selection. The normalised intron excision ratio and graphical presentations were obtained from the GTEx Portal. The ancestral alleles of these SNPs (rs3974604 ancestral-C; rs11722779 ancestral-G; rs71431709 ancestral-A) are associated with higher intron splicing ratios.

**Additional file 5: Table S3**. Pairwise *F*_ST_ analyse of SNPs under positive selection in selected populations (for abbreviation see details in Methods) with different genetic ancestry. A.) *F*_ST_ for SNP under positive selection in all studied populations.B.) *F*_ST_ for SNPs under positive selection in African populations.B.) *F*_ST_ for SNPs under positive selection in European and South Asia populations (*FAMD71D* only in Europeans). C.) *F*_ST_ for SNP under positive selection in East Asia populations.

**Additional file 6: Figure S3.** Violin plot of the eQTL SNP rs10459068 (T/C) of the *PLCZ1* gene. The derived-T allele is associated with increased expression of that gene in the testis tissue. Expression data and the violin plot were obtained from the GTEx Portal.

## Data Availability

The datasets used and/or analysed during the current study are available from the corresponding author on reasonable request.

## Abbreviations

aBSREL: Adaptive Branch-site Random Effects Likelihood
dbGaP: Database of Genotypes and Phenotypes
EHH: Extended Haplotype Homozygosity
eQTLs: Expression Quantitative Trait Loci
GO: Gene Ontology
iHS: Integrated Haplotype Score
LD: Linkage Disequilibrium
LRT: Likelihood Ratio Test
sQTLs: Splicing Quantitative Trait Loci
