# Supplementary material for: Population-specific, recent positive directional selection suggests adaptation of human male reproductive genes to different environmental conditions
Source: BMC Evol Biol. 2020 Feb 13;20:27. doi: 10.1186/s12862-019-1575-0 (PMC7020506; doi:10.1186/s12862-019-1575-0)
Supplement: Supplementary file 2 — Additional file 2: Table S2. Results of the aBSREL analysis with the ω distribution over sites of testis-specific genes in non-human primate branches with test p-value ≤0.05. Given are gene known ontology (GO) terms for the genes. [file 12862_2019_1575_MOESM2_ESM.docx]

**Additional file 2 – Table 2.** Results of the aBSREL analysis with the ω distribution over sites of testis-specific genes in non-human primate branches with test *p*-value ≤ 0.05. Given are gene known ontology (GO) terms for the genes.

| **Species** | **Gene** | **Gene description** | **GO molecular**/**GO biological** | **Test**  ***p-value*** | ***ω* distribution over sites** |
| --- | --- | --- | --- | --- | --- |
| Chimpanzee | *MAGEC2* | *MAGE Family Member C2* | Protein binding GO:0005515  Ubiquitin protein ligase binding GO:0031625  Cellular protein catabolic process GO:0044257 | 0.017 | *ω*1=0.00 (95%)  *ω*2=54.2 (5.2%) |
|  | *RHOXF2* | *Rhox Homeobox Family Member 2* | DNA-binding transcription factor activity, RNA polymerase II-specific GO:0000981  Identical protein binding GO:0005515  Positive regulation of DNA binding GO:0043565  Regulation of transcription by RNA polymerase II GO:0006357 | 0.048 | *ω*1>1000 (100%) |
| Gorilla | *SWT1* | *SWT1 RNA Endoribonuclease Homolog* | Transcription, DNA-templated GO:0006351 | <0.0001 | *ω*1=1.00 (98%)  *ω*2= ∞ (1.6%) |
|  | *TPTE2* | *Transmembrane Phosphoinositide 3-Phosphatase And Tensin Homolog 2* | Phosphatidylinositol-3,4,5-trisphosphate 3-phosphatase activity GO:0016314 Peptidyl-tyrosine dephosphorylation GO:0035335 | 0.0344 | *ω*1=0.00 (88%)  *ω*2=19.5 (12%) |
| Orang-utan | *C9ORF43* | *Chromosome 9 Open Reading Frame 43* | Protein binding GO:0005515 | 0.0006 | *ω*1=1.00 (98%)  *ω*2=∞ (1.8%) |
| Rhesus macaque | *INHA* | *Inhibin Subunit Alpha* | Growth factor activity GO:0008083  Transforming growth factor beta receptor binding GO:0005160  Ovarian follicle development GO:0001541  Positive regulation of follicle-stimulating hormone secretion GO:0046881  Regulation of cell cycle GO:0051726  Regulation of cell proliferation GO:0042127 | 0.041 | *ω*1=0.00 (100%)  *ω*2=358 (0.32%) |
| Olive baboon | *DRICH1* | *Aspartate Rich 1* | Protein binding GO:0005515 | 0.0034 | *ω*1=0.00 (76%)  *ω*2=9.63 (24%) |
|  | *INHA* | *Inhibin Subunit Alpha* | Growth factor activity GO:0008083  Transforming growth factor beta receptor binding GO:0005160  Ovarian follicle development GO:0001541  Positive regulation of follicle-stimulating hormone secretion GO:0046881  Regulation of cell cycle GO:0051726  Regulation of cell proliferation GO:0042127 | <0.0001 | *ω*1=0.00 (97%)  *ω*2>1000 (2.9%) |
|  | *SLF1* | *SMC5-SMC6 Complex Localization Factor 1* | Protein binding GO:0005515  Ubiquitin protein ligase binding GO:0031625 Definition  Cellular response to DNA damage stimulus GO:0006974  Positive regulation of double-strand break repair GO:2000781  Positive regulation of maintenance of mitotic sister chromatid cohesion GO:0034184 | <0.0001 | *ω*1=0.552 (100%)  *ω*2=∞ (0.39%) |
| Common marmoset | *AKAP4* | *A-Kinase Anchoring Protein 4* | Protein binding GO:0005515  Protein kinase A binding GO:0051018  Establishment of protein localization GO:0045184  Flagellated sperm motility GO:0030317 | 0.0012 | *ω*1=0.966 (95%)  *ω*2=15.3 (4.8%) |
|  | *MAGEC2* | *MAGE Family Member C2* | Protein binding GO:0005515  Ubiquitin protein ligase binding GO:0031625  Cellular protein catabolic process GO:0044257 | 0.017 | *ω*1=0.00 (95%)  *ω*2=54.2 (5.2%) |
|  | *SEMG2* | *Semenogelin 2* | Protease binding [GO:0002020](https://www.nextprot.org/term/GO:0002020)  Protein binding GO:0005515  Zinc ion binding GO:0008270  Antibacterial humoral response GO:0019731  Coagulation GO:0050817  Negative regulation of flagellated sperm motility GO:1901318  Positive regulation of serine-type endopeptidase activity GO:1900005  Sperm capacitation GO:0048240 | 0.0075 | *ω*1=2.29 (100%) |
