# Supplementary material for: Population-specific, recent positive directional selection suggests adaptation of human male reproductive genes to different environmental conditions
Source: BMC Evol Biol. 2020 Feb 13;20:27. doi: 10.1186/s12862-019-1575-0 (PMC7020506; doi:10.1186/s12862-019-1575-0)
Supplement: Supplementary file 3 — Additional file 3: Figure S1. EHH plot of the SNP rs11722779 of gene SLC9B1 in the European (TSI) vs. African (LWK) vs population. [file 12862_2019_1575_MOESM3_ESM.docx]

**Additional file 3 – Fig. 1.** EHH plot of the SNP rs11722779 of gene *SLC9B1* in the European (TSI) *vs*. African (LWK) vs population.


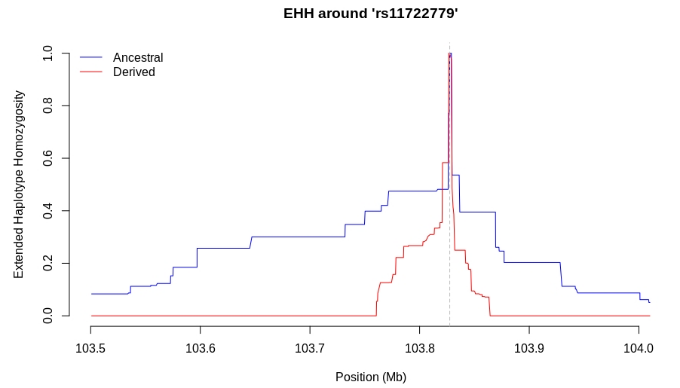

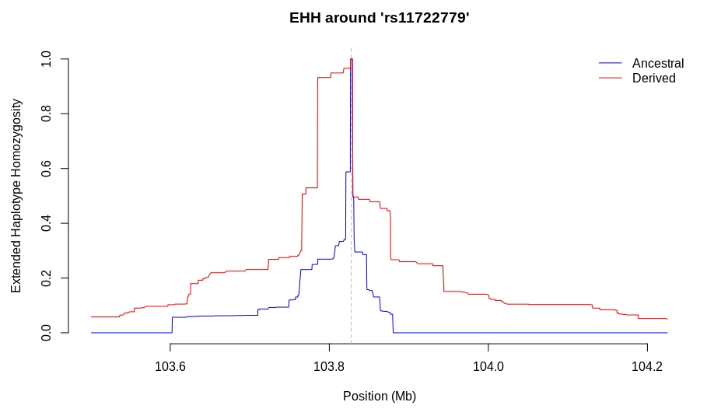
 TSI LWK
