# Supplementary material for: Population-specific, recent positive directional selection suggests adaptation of human male reproductive genes to different environmental conditions
Source: BMC Evol Biol. 2020 Feb 13;20:27. doi: 10.1186/s12862-019-1575-0 (PMC7020506; doi:10.1186/s12862-019-1575-0)
Supplement: Supplementary file 4 — Additional file 4: Figure S2. Violin plots showing the SNP splicing QTLs (sQTLs) of the testis-enriched genes SLC9B1 and RNF17 that are under positive selection. The normalised intron excision ratio and graphical presentations were obtained from the GTEx Portal. The ancestral alleles of these SNPs (rs3974604 ancestral-C; rs11722779 ancestral-G; rs71431709 ancestral-A) are associated with higher intron splicing ratios. [file 12862_2019_1575_MOESM4_ESM.docx]

**Additional file 4 – Fig. 2.** Violin plots showing the SNP splicing QTLs (sQTLs) of the testis-enriched genes *SLC9B1* and *RNF17* that are under positive selection. The normalised intron excision ratio and graphical presentations were obtained from the GTEx Portal. The ancestral alleles of these SNPs (rs3974604 ancestral-C; rs11722779 ancestral-G; rs71431709 ancestral-A) are associated with higher intron splicing ratios.


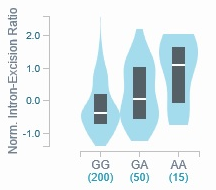


*RNF17* rs71431709


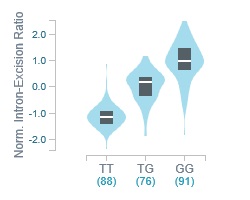

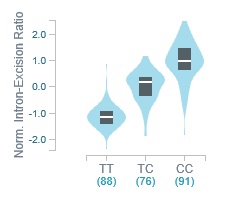


*SLC9B1* rs3974604

*SLC9B1* rs11722779
