# Supplementary material for: Population-specific, recent positive directional selection suggests adaptation of human male reproductive genes to different environmental conditions
Source: BMC Evol Biol. 2020 Feb 13;20:27. doi: 10.1186/s12862-019-1575-0 (PMC7020506; doi:10.1186/s12862-019-1575-0)
Supplement: Supplementary file 5 — Additional file 5: Table S3. Pairwise FST analyse of SNPs under positive selection in selected populations (for abbreviation see details in Methods) with different genetic ancestry. A.) FST for SNP under positive selection in all studied populations.B.) FST for SNPs under positive selection in African populations.B.) FST for SNPs under positive selection in European and South Asia populations (FAMD71D only in Europeans). C.) FST for SNP under positive selection in East Asia populations. [file 12862_2019_1575_MOESM5_ESM.docx]

**Additional File 5 – Table 3**. Pairwise *F*_ST_ analyse of SNPs under positive selection in selected populations (for abbreviation see details in Methods) with different genetic ancestry.

A.) *F*_ST_ for SNP under positive selection in all studied populations.

|  | ***SLC9B1* - rs11722779** | | | |
| --- | --- | --- | --- | --- |
|  | AFR | EUR | SAS | EAS |
|  | **LWK** | **GBR** | **BEB** | **JPT** |
| **LWK** | **-** |  |  |  |
| **GBR** | 0.281 | - |  |  |
| **BEB** | 0.309 | -0.004 | - |  |
| **JPT** | 0.318 | -0.002 | -0.005 | - |

B.) *F*_ST_ for SNPs under positive selection in African populations.

|  | ***MORC1* - rs12695191** | | | |  | ***RNF17* - rs71431709** | | | |  | ***WBP2NL* - rs57796605** | | | |
| --- | --- | --- | --- | --- | --- | --- | --- | --- | --- | --- | --- | --- | --- | --- |
|  | AFR | EUR | SAS | EAS |  | AFR | EUR | SAS | EAS |  | AFR | EUR | SAS | EAS |
|  | **LWK** | **GBR** | **BEB** | **JPT** |  | **LWK** | **GBR** | **BEB** | **JPT** |  | **LWK** | **GBR** | **BEB** | **JPT** |
| **LWK** | **-** |  |  |  |  | **-** |  |  |  |  | **-** |  |  |  |
| **GBR** | 0.312 | - |  |  |  | 0.015 | - |  |  |  | 0.190 | - |  |  |
| **BEB** | 0.317 | -0.004 | - |  |  | 0.055 | 0.005 | - |  |  | 0.186 | nan | - |  |
| **JPT** | 0.351 | 0.007 | 0.001 | - |  | 0.190 | 0.100 | 0.046 | - |  | 0.201 | nan | nan | - |

B.) *F*_ST_ for SNPs under positive selection in European and South Asia populations (*FAMD71D* only in Europeans).

|  | ***DMRT1* - rs166790** | | | |  | ***PLCZ1* - rs10459068** | | | |  | ***FAM71D* - rs10431714** | | | |
| --- | --- | --- | --- | --- | --- | --- | --- | --- | --- | --- | --- | --- | --- | --- |
|  | AFR | EUR | SAS | EAS |  | AFR | EUR | SAS | EAS |  | AFR | EUR | SAS | EAS |
|  | **LWK** | **GBR** | **BEB** | **JPT** |  | **LWK** | **GBR** | **BEB** | **JPT** |  | **LWK** | **GBR** | **BEB** | **JPT** |
| **LWK** | **-** |  |  |  |  | **-** |  |  |  |  | **-** |  |  |  |
| **GBR** | 0.187 | - |  |  |  | 0.342 | - |  |  |  | 0.621 | - |  |  |
| **BEB** | 0.253 | 0.004 | - |  |  | 0.368 | -0.005 | - |  |  | 0.260 | 0.197 | - |  |
| **JPT** | 0.283 | 0.012 | -0.004 | - |  | 0.222 | 0.018 | 0.027 | - |  | 0.156 | 0.291 | 0.013 | - |

C.) *F*_ST_ for SNP under positive selection in East Asia populations

|  | ***ROPN1L* - rs2673855** | | | |
| --- | --- | --- | --- | --- |
|  | AFR | EUR | SAS | EAS |
|  | **LWK** | **GBR** | **BEB** | **JPT** |
| **LWK** | **-** |  |  |  |
| **GBR** | 0.0317133 | - |  |  |
| **BEB** | 0.0277509 | 0.12414 | - |  |
| **JPT** | 0.251582 | 0.39342 | 0.123854 | - |
