# Supplementary material for: Population-specific, recent positive directional selection suggests adaptation of human male reproductive genes to different environmental conditions
Source: BMC Evol Biol. 2020 Feb 13;20:27. doi: 10.1186/s12862-019-1575-0 (PMC7020506; doi:10.1186/s12862-019-1575-0)

**Additional file 6 – Fig. 3.** Violin plot of the eQTL SNP rs10459068 (T/C) of the *PLCZ1* gene. The derived-T allele is associated with increased expression of that gene in the testis tissue. Expression data and the violin plot were obtained from the GTEx Portal.


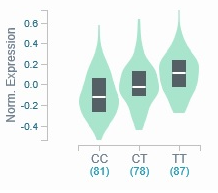

Supplement: Supplementary file 6 — Additional file 6: Figure S3. Violin plot of the eQTL SNP rs10459068 (T/C) of the PLCZ1 gene. The derived-T allele is associated with increased expression of that gene in the testis tissue. Expression data and the violin plot were obtained from the GTEx Portal. [file 12862_2019_1575_MOESM6_ESM.docx]
